# Opioid sparing effect of intravenous dexmedetomidine in orthopaedic surgery: a retrospective analysis

**DOI:** 10.1186/s44158-022-00076-1

**Published:** 2022-12-19

**Authors:** Valerio Donatiello, Aniello Alfieri, Andrea Napolitano, Vincenzo Maffei, Francesco Coppolino, Vincenzo Pota, Maria Beatrice Passavanti, Maria Caterina Pace, Pasquale Sansone

**Affiliations:** 1 Department of Elective Surgery, Postoperative Intensive Care Unit and Hyperbaric Oxygen Therapy, A.O.R.N. Antonio Cardarelli, V.le Antonio Cardarelli 9, 80131 Naples, Italy; 2 Department of Women, Child and General and Specialized Surgery, University of Campania Luigi Vanvitelli, P.zza Miraglia 2, 80138 Naples, Italy

**Keywords:** Locoregional anaesthesia, Dexmedetomidine, Opioid sparing anaesthesia, Sedation

## Abstract

**Background:**

Dexmedetomidine is a highly selective alpha-2 receptor agonist without any effect on the GABA receptor. It provides an excellent sedative and analgesic profile with few side effects. We report our experience with dexmedetomidine use during orthopaedic surgery under locoregional anaesthesia to ensure adequate sedation and optimal postoperative pain control.

**Methods:**

In this retrospective analysis, we included 128 patients who underwent orthopaedic surgery between January 2019 and December 2021. All patients received the same local anaesthetic dose of 20 ml of ropivacaine 0.375% + mepivacaine 0.5% for axillary and supraclavicular block and 35 ml of ropivacaine 0.375% + mepivacaine 0.5% for triple nerve block (femoral, obturator and sciatic nerve). The cohort was divided into two groups based on sedation drugs used during surgery (dexmedetomidine, or group D, vs midazolam, or group M). All patients received postoperative 24-h analgesia consisting of 60 mg of ketorolac, 200 mg of tramadol and 4 mg of ondansetron. The primary outcome measured how many patients in the two groups required an analgesic rescue dose of pethidine and the time to first pethidine administration. To reduce confounding, we included patients in two groups with non-statistically different demo-anamnestic parameters and who received the same dose of intraoperative local anaesthetic and postoperative analgesia.

**Results:**

The number of patients in group D who did not require a rescue dose of analgesia was significantly greater than in group M (49 vs 11, *p* < 0.001). Time-to-first postoperative opioid administration did not show a fundamental difference between the two groups under examination (523.75 ± 131.55 min vs 564 ± 117.84 min). Total opioid consumption was higher in the M group than in the D group (3529.8 ± 30.36 μg vs 1864.8 ± 31.59 μg, *p* 0.075), with a mean opioid consumption significantly higher in the M group than in the D group (26.26 ± 42.8 μg vs 69.21 ± 46.1 μg, *p* < 0.001): D group received 62.06% less opioid than M group.

**Conclusions:**

The continuous infusion of dexmedetomidine during orthopaedic surgery performed under locoregional anaesthesia has been shown to increase the analgesic effect of local anaesthetics and reduce the consumption of major opioids in the postoperative period. Dexmedetomidine offers a unique ability to supply sedation and analgesia without respiratory depression, having a wide safety margin and an excellent sedative capacity. It does not increase the rate of postoperative complications.

## Introduction

Locoregional anaesthesia represents the first anaesthesiology technique in several major surgical procedures. It provides an adequate anaesthesiologic level and an excellent control of intraoperative pain. It also reduces biochemical variations in the postoperative period [1–3], avoiding the risks of general anaesthesia, especially in patients with numerous comorbidities. To ensure an adequate level of procedural sedation and postoperative pain control, opioid drugs are mainly used, burdened by a high complication rate (respiratory and gastrointestinal), especially in elderly patients. Nowadays, opioid-free anaesthesia should be the leading anaesthesiologic standard [4–7]. Postoperative pain treatment (PSPT) plays an increasingly significant role in surgical patient management. PSPT has an estimated average incidence of 30%, depending on the type of surgery [8]. The efficacy of topical local anaesthetics in the treatment of chronic postoperative pain has been proven in the literature [9]. Numerous efforts are underway to search for adjuvant drugs to complement locoregional anaesthesia techniques, trying to enhance the anaesthetic effect and reduce the risks associated with opioid drugs with a high hemodynamic and respiratory impact for postoperative pain control [10–13]. Dexmedetomidine is a highly selective alpha-2 receptor agonist with an excellent sedative and analgesic profile with few side effects [14, 15]; it has been shown to prolong intra- and postoperative analgesia when administered intrathecally and perineurally and in continuous administration [16–18]. In fact, it is widely used in several surgical specialities (gynaecological surgery, cardiac surgery, ophthalmology, etc.) [19, 20] as an adjuvant to locoregional anaesthesia, becoming an alternative to general anaesthesia in high- and medium-risk elderly patients. We, therefore, hypothesised that the administration of dexmedetomidine during orthopaedic surgery under locoregional anaesthesia to ensure adequate sedation may significantly reduce the use of opioids for the control of postoperative pain.

## Methods

In this retrospective analysis, we included a consecutive series of patients who underwent orthopaedic surgery with locoregional anaesthesia (upper limb or lower limb) between January 2019 and December 2021 at AOU “Luigi Vanvitelli”, Naples, Italy. Patients included were (1) aged over 18 years, (2) American Society of Anesthesiologists (ASA) score of less than 3, (3) able to express in words the possible presence of postoperative pain and request the administration of a rescue dose of analgesia, (4) have no contraindications to locoregional anaesthesia technique, and (5) not being under chronic pharmacological treatment with benzodiazepines, opiates, or alpha-2 agonists for pre-existing diseases (anxiety-depressive syndrome, high blood pressure, chronic pain, etc.). All sedation protocols, postoperative pain drug administration and local anaesthetic drug dosage used in locoregional anaesthesia were administered as standard clinical procedures adopted in our hospital. All patients consent to data publication. The consent to data publication for scientific purposes is an integral part of the anaesthetic consent that patients normally sign. The study was conducted in accordance with the International Conference on Harmonisation Good Clinical Practice guidelines and the provisions of the 2008 Declaration of Helsinki. Continuous parameters are reported as frequency, mean, SD, median, first and third quartile, minimum, and maximum. Discrete parameters are reported in tables as count and the related percentage. Baseline homogeneity between the groups was tested by means of the Student *t*-test for continuous parameters. The homogeneity of discrete parameters was tested using the chi-square test with continuity correction if appropriate. All analyses and tables have been produced using SAS version 9.4. Charts have been done using Microsoft Excel 2016.

## Results

A total of 122 patients have been identified. Of these, 71 had received dexmedetomidine as sedation, and 51 had received midazolam as sedation. The mean age of patients in the D group was 47.5 ± 22.9 years, with twenty-nine men and forty-two women. The mean age of patients in the M group was 49.8 ± 22.3 years, with twenty-one men and thirty women (Tables 1 and 2). There is not any statistical difference between the study groups as concern age, gender (59% of this sample are females) and BMI. The two study groups are not statistically different regarding the demo-anamnestic parameters (age, sex, BMI and surgical time). Due to this result, matching the enrolled subjects between the study groups is not necessary. All patients received the same local anaesthetic dose: 20 ml of ropivacaine 0.375% (75 mg) + mepivacaine 0.5% (100 mg) for axillary and supraclavicular block and 35 ml of ropivacaine 0.375% (131.25 mg) + mepivacaine 0.5% (175 mg) for triple nerve block (femoral, obturator and sciatic nerve), as a standard procedure adopted in our hospital. We only selected patients in whom peripherical nerve block was successful and did not require a deeper anaesthesiologic plan to perform surgery. The cohort was then divided into two groups based on the sedation drug used during surgery, and the sedation level was assessed using the modified Ramsay Sedation Score Scale (mRSS). Awake levels were as follows: 1, patient anxious and agitated and/or restless; 2, patient cooperative, orientated, and tranquil; and 3, patient responds to commands only. Asleep levels depended on the patient’s response to a light glabellar tap or loud auditory stimulus, with levels 4–6 indicating a brink response, sluggish response and no response, respectively. Group M received midazolam 2 mg (or 3 mg if body mass weight > 60 kg) with a rescue dose of 2 mg if mRSS < 2, and group D received dexmedetomidine 1 μg/kg/h for the first 20 min, followed by an infusion of 0.5–0.8 μg/kg/h to maintain an mRSS > 2. Locoregional anaesthesia techniques included supraclavicular brachial plexus block, axillary brachial plexus block, and femoral, obturator and sciatic nerves block (triple nerve block), using a Sonosite SII with HFL50x 15–6 MHz linear probe. In group D, 22 patients received axillary brachial plexus block, 12 patients received supraclavicular brachial plexus block and 37 patients received triple nerve block. In group M, 17 patients received axillary brachial plexus block, 9 patients received supraclavicular brachial plexus block and 25 patients received triple nerve block. All patients received postoperative 24-h analgesia consisting of 60 mg of ketorolac, 200 mg of tramadol and 4 mg of ondansetron as standard postoperative pain control therapy. We analysed the request for a rescue dose of opioids for postoperative pain control by administering pethidine 50 mg i.m. The primary outcome measured how many patients in the two groups required an analgesic rescue dose of pethidine and the time to the first pethidine administration. The total opioid dose consumed in the two groups was calculated by converting 25 mg of pethidine into 33.3 μg of fentanyl. During the intraoperative period, vital and haemodynamic parameters (SpO2, FC, PA) were noted and compared to demonstrate the tolerability of dexmedetomidine, and any adverse reactions were evaluated and treated. In particular, the following were examined: bradycardia (< 45 bpm) treated with administration of 0.5 mg of atropine, hypotension (< 80 mmHg of systolic pressure) treated with 2 mg of ephedrine, desaturation (SpO_2_ < 90%) treated with administration of 4 L/min of O_2_, postoperative nausea and vomiting (PONV), headache and dizziness. Comparing the study groups, the percentage of subjects who required a rescue dose of analgesic is still statistically (*P* < 0.01) different (31% in the dexmedetomidine group and 78.4% in the midazolam group) (Table 3). On the other hand, time to the first pethidine administration did not show a fundamental difference between the two groups under examination (523.75 ± 131.5 min vs 564 ± 117.8 min). Total opioid consumption was increased in the M group than in the D group (3529.8 μg vs 1864.8 μg), with a mean opioid consumption significantly higher in the M group than in the D group (26.26 ± 42.8 μg vs 69.21 ± 46.1 μg, *P* < 0.001): D group received 62.06% less opioid than M group (Table 4). Nine episodes of postoperative dizziness occurred in group M (17.64%, *P* < 0.001) (Table 5).

**Table 1:** Demographic parameters of the study population

Parameters	Statistics	Dex (***N*** = 71)	Mid (***N*** = 51)	All subjects (***N*** = 122)
Age (years)	*n*	71	51	122
	Mean	47.5 (± 22.9)	49.8 (± 22.3)	48.4 (± 22.5)
	Median	47	53	51.5
	Q1 ÷ Q3	26.0 ÷ 66.0	26.0 ÷ 67.0	26.0 ÷ 66.0
	Min ÷ max	16 ÷ 97	18 ÷ 92	16 ÷ 97
Sex				
Male	*n* (%)	29 (40.8)	21 (41.2)	50 (41.0)
Female	*n* (%)	42 (59.2)	30 (58.8)	72 (59.0)

**Table 2:** Anamnestic parameters of the study population

Parameters	Statistics	Dex (***N*** = 71)	Mid (***N*** = 51)	All subjects (***N*** = 122)
BMI (kg/m^2^)	Mean	25.5 (± 3.0)	24.8 (± 2.4)	25.2 (± 2.8)
	Median	25	24	25
	Q1 ÷ Q3	23.0 ÷ 27.0	23.0 ÷ 26.0	23.0 ÷ 26.0
	Min ÷ max	21 ÷ 37	21 ÷ 37	21 ÷ 37
Surgical time (min)	Mean	95.4 (± 33.0)	100.9 (± 21.7)	97.7 (28.9)
	Median	93	103	96
	Q1 ÷ Q3	73.0 ÷ 109.0	86.0 ÷ 115.0	82.0 ÷ 109.0
	Min ÷ max	39 ÷ 210	68 ÷ 165	39 ÷ 210

**Table 3:** Primary endpoint evaluated in the study groups matched with the Mahalanobis distance technique

Parameters	Dex (***N*** = 71)	Mid (***N*** = 51)	***P***-value
Rescue medication			
Yes	22 (31%)	40 (78.4%)	< 0.0001
No	49 (69%)	11 (21.6%)	< 0.01

**Table 4:** Opioid consumption and time to the first request of an analgesic in the study population

Parameters	Dex (***N*** = 71)	Mid (***N*** = 51)	***P***-value
Total opioid consumption (μg)	1864.8 (± 31.59)	3529.8 (± 30.36)	0.075
Mean opioid consumption (μg/person)	26.26 (± 42.8)	69.21 (± 46.1)	< 0.0001
Time to the first request of analgesic (min)	523.7 (± 131.5)	564 (± 117.8)	0.084

**Table 5:** Adverse effect on the study population

Parameters	Dex (***N*** = 71)	Mid (***N*** = 51)	***P***-value
Bradycardia	2 (2.8%)	0	0.217
Hypotension	0	1 (1.97%)	0.237
Desaturation	0	1 (1.97%)	0.237
PONV	2 (2.8%)	1 (1.97%)	0.770
Headache	1 (1.4%)	0	0.401
Dizziness	0	9 (17.6%)	< 0.001

## Discussion

The main findings of this study are (1) continuous intravenous administration of dexmedetomidine reduces opioid consumption in the postoperative period, (2) prolongs analgesic duration of local anaesthetic and (3) showed an excellent tolerability profile.

International literature has paid close attention to the role of bolus dexmedetomidine administered together with a local anaesthetic. Still, our analysis suggests the importance of continuous intravenous administration to maintain long-term analgesia [16–20]. The pharmacodynamic role of dexmedetomidine on the alpha-2 receptors in maintaining and prolonging analgesic efficacy is therefore evident [14, 15]. There are several mechanisms involved thanks to which dexmedetomidine extends nerve block, including local vasoconstriction, systemic effect and direct action on the nerve and on spinal and supraspinal levels. Intravenous dexmedetomidine seems to be more effective thanks to the direct action on the alpha 2 adrenoreceptors present in the locus coeruleus. In addition, intravenous administration of dexmedetomidine produces a greater degree of differential block on the A-δ and unmyelinated C fibres involved in the modulation of sensory conduction compared to the A-α fibres involved in motor conduction. For the above reasons, it is possible to achieve excellent pain control by sparing motor function in the postoperative period.

Midazolam shows no prolonged effects after systemic administration, while dexmedetomidine sedation was prolonged, no matter which injection route was chosen [17]. Dexmedetomidine also reduced 24-h cumulative opioid consumption; IV was non-inferior to perineural for these outcomes. In our present study, a sedation dose of dexmedetomidine prolonged the duration of analgesia on both upper and lower limb blocks. Total opioid consumption was significantly lower in group D rather than in group M. Our analysis confirms that sedation via intravenous dexmedetomidine can positively affect analgesia during locoregional anaesthesia [16–20]. Our analysis confirms the excellent tolerability profile of both sedatives, as already demonstrated by literature and which gives ever greater space to the use of dexmedetomidine even outside the management of patients in critical areas [17]. Another advantage of dexmedetomidine sedation is the reduction of dizziness. We did observe a significant difference between the groups; no dizziness cases were registered in the D group, while nine patients in the M group manifested dizziness at the end of surgery. However, despite the direct antiemetic effect of the alpha 2 agonist, with reduction of nausea and vomiting due to sympathetic tone depression, and reduced opioid usage, no significant difference in PONV was observed between the two groups. While our results reveal a significant reduction in opioid consumption, it may not lead to significant changes in PONV since postoperative pain is relatively mild after surgery in our patient groups, even without additional dexmedetomidine administration. Hong et al. demonstrated a significant difference in time-to-first in patients undergoing brachial plexus blockade sedated with dexmedetomidine vs midazolam [16]. However, our analysis shows no real difference between the groups. In our research, following the administration of a loading dose of 1 μg/kg/h of dexmedetomidine, patients were administered 0.5–0.8 μg/kg/h of dexmedetomidine for sedation. Even in patients with renal disease, doses of dexmedetomidine are adjusted to 0.2–0.7 μg/kg/h to maintain RSS scores of 3–4 after a 1-μg/kg/h loading dose. The relatively higher doses of dexmedetomidine administered to those patients resulted in no respiratory depression or desaturation events. It is well known that dexmedetomidine causes minimal depression of respiratory drive [14]. Dexmedetomidine deepens the level of sedation in a dose-dependent manner. Reducing opioid use in the postoperative period may be the winning strategy for reducing the incidence of postoperative chronic pain [8]. Although this study demonstrated an effective reduction in postoperative opioid demand, it has several limitations, primarily that of being a retrospective analysis. It is also difficult to assess the relationship between the intravenous dose of dexmedetomidine and the duration of analgesia because each patient received a different dose of sedative (0.5–0.8 μg/kg/h). Moreover, although dexmedetomidine showed a greater affinity for sensory block, no data on the quality of motor and sensory functions were collected.

## Conclusions

The continuous infusion of dexmedetomidine during orthopaedic surgery performed under locoregional anaesthesia has been shown to increase the analgesic effect of local anaesthetics and reduce the consumption of major opioids in the postoperative period and avoid, however, devices not easy to use [21, 22]. Dexmedetomidine offers a unique ability to supply sedation and analgesia without respiratory depression, having a wide safety margin and an excellent sedative capacity. It does not increase the rate of postoperative complications. In conclusion, sedation with dexmedetomidine in orthopaedic surgery may be a viable alternative for reducing postoperative opioid use in elderly patients.

## Data Availability

The datasets generated during and/or analysed during the current study are available from the corresponding author upon reasonable request.
